# A descriptive study of the clinico-pathological and surgical characteristics of patients with primary epithelial ovarian cancer. A cross sectional study

**DOI:** 10.1016/j.amsu.2020.09.043

**Published:** 2020-10-02

**Authors:** Amer Sindiani, Basil Obeidat, Leen Hamadeh, Shahed Alghazo, Alia Al-Mohtaseb, Eman Alshdaifat

**Affiliations:** a Department of Obstetrics and Gynecology, Jordan University of Science and Technology, Irbid, Jordan; b Department of Pathology and Microbiology, Jordan University of Science and Technology, Irbid, Jordan; c Department of Obstetrics and Gynecology, Yarmouk University, Jordan

**Keywords:** Primary epithelial ovarian cancer, Ovarian cancer, Clinico-pathology

## Abstract

**Objective:**

To study the clinical, pathological and surgical features of primary epithelial ovarian cancer treated at our institution.

**Methods:**

fifty-nine patients with primary epithelial ovarian cancer were included. Clinical data collected included patient's age, presenting symptoms, laboratory and tumor markers results as well as preoperative imaging reports. Pathological and surgical findings included were: spread of the disease, histologic type, stage of the disease, type of surgical procedure and amount of residual disease.

**Results:**

Mean age of the patients was 54.5 years. Lower abdominal pain was the most common presenting symptom, followed by abdominal distension. The commonest histopathological type was high grade serous carcinoma (72.9%). In our study, majority of patients were diagnosed with stage III disease, accounting for 69.5% of the total number of patients. Complete cytoreduction with no gross residual disease was achieved in 77.3% of patients with stage 3–4 disease.

**Conclusion:**

clinical and pathological features of primary epithelial ovarian carcinoma in our populations are similar to what is reported worldwide. We have also documented that our surgical approach to the management of ovarian cancer is comparable to the international consensus.

## Highlights

• The commonest histopathological type of ovarian cancer in our institute is high grade serous carcinoma (72.9%).
• In our study, majority of patients were diagnosed with stage III disease, accounting for 69.5% of the total number of patients.
• Complete cytoreduction with no gross residual disease was achieved in 77.3% of patients with stage 3–4 disease.
• We have reported a high complete cytoreduction rate (77.3%).

## 1 Introduction

Epithelial ovarian cancer (EOC) is the major cause of gynecological cancer-related mortality in developed countries, with annual incidence of more than 200,000 new cases and responsible of 150,000 deaths worldwide [1]. Due to its subtle symptomatology and the lack of specific screening methods, about 70% of EOCs are diagnosed in advanced stage, specifically International Federation of Gynecology and Obstetrics (FIGO) stage III and IV [2]. Advanced-stage ovarian cancer has a 5-year survival rate of 30–55%, while early stages have a 5-year survival of over 80% [3].

Serum CA-125, a tumor marker for ovarian cancer, has been observed to be elevated in 80% of women with epithelial ovarian cancer overall, but in only 50% of women with early disease [4].

There is little doubt that early stage ovarian cancer is significantly more curable than late stage disease. An early detection of ovarian cancer and timely reference to gynecologic oncologist is the milestone to reduce the mortality from ovarian cancer. Several diagnostic methods for pelvic mass have been reported such as pelvic examination, ultrasonography, CA-125 tumor marker level. However, none of these methods used individually has shown significantly better performance in detecting malignant ovarian tumor. Hence, this led to the development of a mathematical formula using a combination of these diagnostic modalities to predict whether an adnexal mass is benign or malignant - risk of malignancy index (RMI). RMI was originally developed in 1990, and it was termed RMI 1 [5]. This index was defined as the product of menopausal score (M), ultrasound score (U), and the absolute value of serum CA-125 level and reported a sensitivity of 85.4% and specificity of 96.9% at the cutoff value of 200. The best cutoff value of RMI for the distinction between benign and malignant masses has been proved to be 200 [5,6].

Except for patients not eligible for surgery due to severe comorbidities or extensive tumor spread, the standard treatment for advanced stage EOC is primary debulking surgery (PDS), with the goal of optimal cytoreduction followed by adjuvant chemotherapy with paclitaxel plus platinum based agents [2,7]. Survival in patients affected by EOC is strongly related to the residual disease after surgical treatment [2]. Patients without macroscopic residual tumor (complete debulking) showed a better survival than patients with minimal residual disease < 1 cm (optimal debulking) and patients with residual disease > 1 cm (suboptimal debulking) [8]. The possibility to attain complete cytoreduction depends on several factors like the spread of the disease, the molecular features of the tumor, its microenvironment and the skill of gynecologic oncology surgeon [9,10].

For patients in whom a complete cytoreduction during primary surgery is not expected, neoadjuvant chemotherapy (NACT) followed by interval debulking surgery (IDS) is considered the most appropriate therapeutic option [2,11]. Recent studies demonstrated that such strategy allows higher rate of no residual disease in comparison to primary surgery [[12], [13], [14]]. Two randomized controlled studies have been published in order to compare survivals of PDS versus NACT + IDS strategy. Both EORTC and the most recent CHORUS trial showed no differences in overall survival (OS) and progression free survival (PFS) in the two treatments arms [15,16].

Little is known about the ovarian cancer presentation, behavior and management in Jordan. Therefore, the aim of this study was to describe the clinical and pathological features of epithelial ovarian cancer in our community and to evaluate the surgical treatment of such patients.

## 2 Materials and methods

### 2.1 Design and settings

Between 2010 and 2019, we retrospectively studied all cases of primary epithelial ovarian cancer operated at King Abdullah University Hospital.

### 2.2 Sample

We reviewed the patient's individual records as well as the pathology reports. Clinical data collected included patient's age, presenting symptoms, laboratory and tumor markers results as well as preoperative imaging reports. Risk of malignancy index was calculated according to Jacobs et al. equation [5].

Pathological and surgical findings include: spread of the disease, histologic type, stage of the disease, type of surgical procedure and amount of residual disease. Ovarian cancers were staged according to the International Federation of Gynecology and Obstetrics system of classification [17]. Recurrent ovarian cancers, and cases considered as primary peritoneal carcinoma were excluded.

### 2.3 Ethical considerations

Institutional Review Board was obtained (Ref. Number: 170/132/2020) and according to Helsinki declaration the research is registered in research registry database (researchregistry5892).

### 2.4 Statistical analysis

Statistical analysis was performed using The Statistical Package for Social Sciences (SPSS) 17.0 for Microsoft Windows. A STROCSS guidelines used to report our study [35].

## 3 Results

During the study period, a total of fifty-nine cases were included and were available for analysis.

The mean age of the patients was 54.5 (range 27–74 years). Thirty-nine patients (66.1%) were postmenopausal.

Ovarian cancer usually presents with nonspecific symptoms resulting in patients seeking medical help in advances stages. It has been very difficult to define the symptoms attributable to ovarian cancer. In our study, the majority of patients presented with lower abdominal pain (50.1%), followed by abdominal distension – reported by 19 (32.2%%) patients. Around 16 patients (27%) reported nonspecific symptoms. In the study group, nine (15.3%) patients reported postmenopausal bleeding (exclusion of other causes was done). However, 23.7% of cases were diagnosed during work up for non-gynecological presentation.

Ca125 level was elevated (above 35 IU/ml) in 93.2% of cases. Risk of malignancy index (RMI) was above cutoff value of 200 in 84.7% of cases. In this study, the commonest histopathological type was high grade serous carcinoma (72.9%). Clear cell, mucinous and endometrioid types were found in 6.8%, 5.1% and 3.3% respectively, as shown in Table 1.

**Table 1:** Patient characteristics (59 patients).

	N (%)
**All patients**	59
**Age**	54.5 (27–74)
**Symptoms at diagnosis**	
- Non specific	16 (27%)
- Distension	19 (32.2%)
- Abdominal pain	30 (50.1%)
- Post-menopausal bleeding	9 (15.3%)
- Work up for ascites or pleural effusion	14 (23.7%)
**Ca125 level**	822 (12.3–5600)
- Elevated level >35 iu/ml	55 (93.2%)
**Risk malignancy index (RMI)**	6537 (36.9–50526)
- RMI > 200	50 (84.7%)
**Histopathology type**	
- High grade serous	43 (72.9%)
- Low grade serous	5 (8.5%)
- Clear cell	4 (6.8%)
- Mucinous	3 (5.1%)
- Endometrioid	2 (3.3%)
- Transitional	1 (1.7%)
- Carcinosarcoma	1 (1.7%)

Pelvic organs and peritoneum was the most site of involvement by ovarian cancer (80.3%). Outside the pelvis, omentum was the most common site of gross involvement (59.3%). Ascites was found in 38 patients (64.4%) of cases. In our study, majority of patients were diagnosed with stage III disease, accounting for 69.5% of the total number of patients. Of the 59 cases, 5.1% were reported to be in the stage IV of the disease (Table 2).

**Table 2:** Surgical outcome of patients.

	N (%)
**Disease site**	
- Pelvic	48 (81.3%)
- Ascites	38 (64.4%)
- Omentum	35 (59.3%)
- Peritoneum	29 (49.2%)
- Bowel (serosa, mesentery)	20 (33.4%)
- Liver surface	2 (3.3%)
**Stage of disease**	
- Stage I	12 (20.3%)
- Stage II	3 (5.1%)
- Stage III	41 (69.5%)
- Stage IV	3 (5.1%)
**Type of surgery(all patients** = **59)**	
- Primary debulking surgery	32 (54.2%)
- Interval debulking surgery	27 (45.8)
- Bowel resection	4 (6.8%)
**Type of surgery (stage III-IV, N** = **44)**	
- Primary debulking surgery	18 (40.9%)
- Interval debulking surgery	26 (59.1%)
**Residual disease**	
- No residual disease	48(81.3%)/34(77.3%) stage 3–4
- Any size residual disease	11(18.7%)/10(22.7%) stage 3–4

In our study, patients were managed either by primary debulking surgery (PDS) followed by chemotherapy or by neoadjuvant chemotherapy followed by interval debulking surgery (NACT and IDS). Among all patients, primary surgery was performed in 54.2% of cases. However, only 40.9% of patients with advanced disease (stage 3 and 4) underwent primary surgery, while the remaining 59.1% patients had NACT and IDS. Complete cytoreduction with no gross residual disease was achieved in 77.3% of patients with stage 3–4 disease, compared to 81.3% among all patients.

In patients with stage III-IV disease, the rate of optimal cytoreduction with no residual disease was significantly higher in the group managed by NACT and IDS than those treated by primary surgery. However, age, ca125 level and RMI were not statistically predictive of optimal cytoreduction (Table 3).

**Table 3:** Residual disease in patients with stage III-IV disease.

	Any residual disease (N = 10)	No residual disease (N = 34)	*P*- Value
**Type of surgery**			
- Primary debulking	7	11	0.0332
- Interval debulking	3	23	
Average age (years)	53.4	58.1	NS
Ca125 level (iu/ml)	1206.7	796.2	NS
RMI	8126.1	6563.7	NS

There was no significant difference in complications between the two different modality of treatment.

## 4 Discussion

Ovarian cancer accounts for more deaths than all other gynecologic malignancies combined [1]. However, there are no effective screening tests for ovarian cancer and few notable early symptoms.

According to the American Cancer Society, approximately 90% of ovarian cancers are epithelial ovarian carcinomas which are subdivided based on their microscopic features into serous, mucinous, endometrioid, clear cell, and undifferentiated [18]. Age has a strong correlation to ovarian cancer risk and 64% of cases are diagnosed after 50 years of age [19]. An epidemiological risk prediction model by Morice et al. reported a median age of EOC in various countries as 52.4 years [20]. In our study, the mean age of the patients was 54.5 years and 66.1% were postmenopausal. This is in agreement with literature.

In our study, the most common presenting symptom was abdominal pain, reported by 50% of patients. But in a study conducted by Kate E Brain [21] et al., in 2014, the most common presenting complaints were post menopausal bleeding, pelvic and abdominal pain which was around 87%. Other studies reported similar symptoms and presentations to our data [22]. This reflects the diversity and failure to recognize symptoms of early disease.

Different histologic subtypes of epithelial ovarian cancer have been described. In our study, serous carcinoma was the predominant type, particularly in advanced stages. This is in concordance with reported in the literature [23,24].

The disease is diagnosed in late stages as there is a delay between the onset of symptoms and diagnosis [25]. Several authors reported that 60–70% of cases were diagnosed in Stage 3 and 4 [2,26]. Our data showed similar results where about 74% of cases were diagnosed at advanced stage.

Several diagnostic methods for pelvic mass have been reported such as pelvic examination, ultrasonography, CA-125 tumor marker level. However, none of these methods used individually has shown significantly better performance in detecting malignant ovarian tumor. The risk of malignancy index (RMI) incorporating CA 125, menopausal status and ultrasound was consistently found to be effective method of discriminating between ovarian cancer and benign lesions preoperatively [5,6]. At cutoff value of 200, RMI has a sensitivity of 84.6% for predicting ovarian cancer in our study which is consistent with previous similar studies [5,6,27].

It is now accepted that the goal of cytoreductive surgery should be complete resection of macroscopically visible tumor [28,29]. However, in advanced stage EOC the rate of complete debulking is generally estimated lower than 50% [[30], [31], [32]]. The reasons for suboptimal debulking may be related to large intra-abdominal extension of the tumor, localization in critical anatomi-cal site, medical comorbidities, advanced age and poor oncological experience of surgeons. All such variables are of crucial importance to understand the reasons that led the paradigm shift, in selected cases, from standard PDS approach to alternative therapeutic options like NACT + IDS treatment [33,34]. We have reported a high complete cytoreduction rate (77.3%). This is explained by the fact that we have a higher rate of NACT administration before surgery in our study. This is consistent with similar reports in the literature [33,34].

## 5 Conclusion

Despite the limitation of our study (being retrospective in nature and small sample size), we have shown that clinical and pathological features of primary epithelial ovarian carcinoma in our populations are similar to what is reported worldwide. Moreover, we have also documented that our surgical approach to the management of ovarian cancer is comparable to the international consensus. However, it would be of paramount importance to see how this is reflected on patient survival.

## Funding

No funding.

## Provenance and peer review

Not commissioned, externally peer reviewed.

## Declaration of competing interest

No conflict of interest.

## References

[1] Siegel R.L., Miller K.D., Jemal A. (2017). Cancer statistics, 2017. Ca - Cancer J. Clin..

[2] Ataseven B., Chiva L.M., Harter P., Gonzalez-Martin A., du Bois A. (2016). FIGO stage IV epithelial ovarian, fallopian tube and peritoneal cancer revisited. Gynecol. Oncol..

[3] Romanidis K., Nagorni E.A., Halkia E., Pitiakoudis M. (2014 Jul-Sep). The role of cytoreductive surgery in advanced ovarian cancer: the general surgeon's perspective. J BUON.

[4] Kauff N.D., Satagopan J.M., Robson M.E., Offit K. (2002). Risk reducing salpingo-oophorectomy in women with a BRCA1 or BRCA2 mutation. N. Engl. J. Med..

[5] Jacobs I., Oram D., Fairbanks J., Turner J., Frost C., Grudzinskas J.G. (1990). A risk of malignancy index incorporating CA 125, ultrasound and menopausal status for the accurate preoperative diagnosis of ovarian cancer. Br. J. Obstet. Gynaecol..

[6] Obeidat B.R., Amarin Z.O., Latimer J.A., Crawford R.A. (2004 Jun). Risk of malignancy index in the preoperative evaluation of pelvic masses. Int. J. Gynaecol. Obstet..

[7] Kumar L., Pramanik R., Kumar S., Bhatla N., Malik S. (2015). Neoadjuvant chemotherapy in gynaecological cancers - implications for staging. Best Pract. Res. Clin. Obstet. Gynaecol..

[8] du Bois A., Reuss A., Pujade-Lauraine E., Harter P., Ray-Coquard I., Pfisterer J. (2009 15). Role of surgical outcome as prognostic factor in advanced epithelial ovarian cancer: a combined exploratory analysis of 3 prospectively randomized phase 3 multicenter trials: by the Arbeitsgemeinschaft Gynaekologische Onkologie Studiengruppe Ovarialkarzinom (AGO-OVAR) and the Groupe d'Investigateurs Nationaux Pour les Etudes des Cancers de l'Ovaire (GINECO). Cancer.

[9] Kessous R., Laskov I., Abitbol J., Bitharas J., Yasmeen A., Salvador S. (2017). Clinical outcome of neoadjuvant chemotherapy for advanced ovarian cancer. Gynecol. Oncol..

[10] Liu Z., Beach J.A., Agadjanian H., Jia D., Aspuria P.J., Karlan B.Y. (2015). Suboptimal cytoreduction in ovarian carcinoma is associated with molecular pathways characteristic of increased stromal activation. Gynecol. Oncol..

[11] Melamed A., Hinchcliff E.M., Clemmer J.T., Bregar A.J., Uppal S., Bostock I. (2016). Trends in the use of neoadjuvant chemotherapy for advanced ovarian cancer in the United States. Gynecol. Oncol..

[12] May T., Comeau R., Sun P., Kotsopoulos J., Narod S.A., Rosen B. (2017). A comparison of survival outcomes in advanced serous ovarian cancer patients treated with primary debulking surgery versus neoadjuvant chemotherapy. Int. J. Gynecol. Canc..

[13] Mueller J.J., Zhou Q.C., Iasonos A., O'Cearbhaill R.E., Alvi F.A., El Haraki A. (2016). Neoadjuvant chemotherapy and primary debulking surgery utilization for advanced-stage ovarian cancer at a comprehensive cancer center. Gynecol. Oncol..

[14] Rosen B., Laframboise S., Ferguson S., Dodge J., Bernardini M., Murphy J. (2014). The impacts of neoadjuvant chemotherapy and of debulking surgery on survival from advanced ovarian cancer. Gynecol. Oncol..

[15] Kehoe S., Hook J., Nankivell M., Jayson G.C., Kitchener H., Lopes T. (2015). Primary chemotherapy versus primary surgery for newly diagnosed advanced ovarian cancer (CHORUS): an open-label, randomised, controlled, non-inferiority trial. Lancet.

[16] Vergote I., Tropé C.G., Amant F., Kristensen G.B., Ehlen T., Johnson N. (2010). European organization for research and treatment of cancer- gynaecological cancer group; NCIC clinical trials group. Neoadjuvant chemotherapy or primary surgery in stage IIIC or IV ovarian cancer. N. Engl. J. Med..

[17] Prat J., FIGO Committee on Gynecologic Oncology (2014). Staging classification for cancer of the ovary, fallopian tube, and peritoneum. Int. J. Gynaecol. Obstet..

[18] Feeley K.M., Wells M. (2001). Precursor lesions of ovarian epithelial malignancy. Histopathology.

[19] Kurman R.J., Shih IeM. (2016). The dualistic model of ovarian carcinogenesis: revisited, revised, and expanded. Am. J. Pathol..

[20] Morice P., Uzan C., Fauvet R., Gouy S., Duvillard P., Darai E. (2012). Borderline ovarian tumour: pathological diagnostic dilemma and risk factors for invasive or lethal recurrence. Lancet Oncol..

[21] Kate E Brain, Smits Stephanie, Alice E Simon (2014). Ovarian cancer symptoms awareness amd anticipated delayed presentation in a population sample. BMC Canc..

[22] Lataifeh I., Marsden D.E., Robertson G., Gebski V., Hacker N.F. (2005 Jun). Presenting symptoms of epithelial ovarian cancer. Aust. N. Z. J. Obstet. Gynaecol..

[23] Rubin Stephen C., Benjamin Ivor, Kian Md (2014). Clinical and pathological features of ovarian carcinoma in women with germline mutations of BRCA1. N. Engl. J. Med..

[24] Akakpo Patrick Kafui, Derkyi-Kwarten Leonard, Gyasi Richard Kwasi (2017). A pathological and clinical study of 706 primary tumours of the ovary in the largest tertiary hospital in Ghana, Akakpo et al. BMC Wom. Health.

[25] Long B., Chang J., Ziogas A., Tewari K.S., Anton-Culver H., Bristow R.E. (2015). Impact of race, socioeconomic status, and the health care system on the treatment of advanced-stage ovarian cancer in California. Am. J. Obstet. Gynecol..

[26] Bristow R.E., Powell M.A., Al-Hammadi N., Chen L., Miller J.P., Roland P.Y. (2013). Disparities in ovarian cancer care quality and survival according to race and socioeconomic status. J. Natl. Cancer Inst..

[27] Tingulstad S.1, Hagen B., Skjeldestad F.E., Onsrud M., Kiserud T., Halvorsen T., Nustad K. (1996 Aug). Evaluation of a risk of malignancy index based on serum CA125, ultrasound findings and menopausal status in the pre-operative diagnosis of pelvic masses. Br. J. Obstet. Gynaecol..

[28] Karam A., Ledermann J.A., Kim J.W., Sehouli J., Lu K., Gourley C. (2017). Fifth ovarian cancer consensus conference of the gynecologic cancer InterGroup: first-line interventions. Ann. Oncol..

[29] Chang S.J., Hodeib M., Chang J., Bristow R.E. (2013). Survival impact of complete cytoreduction to no gross residual disease for advanced-stage ovarian cancer: a meta-analysis. Gynecol. Oncol..

[30] May T., Comeau R., Sun P., Kotsopoulos J., Narod S.A., Rosen B. (2017). A comparison of survival outcomes in advanced serous ovarian cancer patients treated with primary debulking surgery versus neoadjuvant chemotherapy. Int. J. Gynecol. Canc..

[31] Mueller J.J., Zhou Q.C., Iasonos A., O'Cearbhaill R.E., Alvi F.A., El Haraki A. (2016). Neoadjuvant chemotherapy and primary debulking surgery utilization for advanced-stage ovarian cancer at a comprehensive cancer center. Gynecol. Oncol..

[32] Rosen B., Laframboise S., Ferguson S., Dodge J., Bernardini M., Murphy J. (2014). The impacts of neoadjuvant chemotherapy and of debulking surgery on survival from advanced ovarian cancer. Gynecol. Oncol..

[33] Cornelis S., Van Calster B., Amant F., Leunen K., van der Zee A.G., Vergote I. (2012). Role of neoadjuvant chemotherapy in the management of stage IIIC-IV ovarian cancer: survey results from the members of the European Society of Gynecological Oncology. Int. J. Gynecol. Canc..

[34] Dewdney S.B., Rimel B.J., Reinhart A.J., Kizer N.T., Brooks R.A., Massad L.S. (2010). The role of neoadjuvant chemotherapy in the management of patients with advanced stage ovarian cancer: survey results from members of the Society of Gynecologic Oncologists. Gynecol. Oncol..

[35] Agha R., Abdall-Razak A., Crossley E. (2019). STROCSS 2019 Guideline: strengthening the reporting of cohort studies in surgery. Int. J. Surg..

